# A cross-sectional study on the association between major dietary pattern and impaired fasting glucose

**DOI:** 10.3389/fnut.2025.1521571

**Published:** 2025-03-25

**Authors:** Sara Shojaei-Zarghani, Mohammad Reza Fattahi, Asma Kazemi, Nastaran Najafi, Ali Reza Safarpour

**Affiliations:** ^1^Colorectal Research Center, Shiraz University of Medical Sciences, Shiraz, Iran; ^2^Gastroenterohepatology Research Center, Shiraz University of Medical Sciences, Shiraz, Iran; ^3^Nutrition Research Center, Shiraz University of Medical Sciences, Shiraz, Iran; ^4^Patient Safety Research Center, Clinical Research Institute, Urmia University of Medical Sciences, Urmia, Iran

**Keywords:** glucose intolerance, dietary patterns, healthy diet, Western diet, carnivorous diet

## Abstract

**Background:**

Impaired fasting glucose (IFG) is a precursor to type 2 diabetes and is influenced by dietary factors. This cross-sectional study assessed the association between major dietary patterns and IFG in the baseline phase of PERSIAN Kavar cohort study (PKCS).

**Methods:**

The study included 3,144 participants aged 35–70 years. After assessing dietary intakes by a food frequency questionnaire, principal component analysis was used to identify dietary patterns. Logistic regression model was applied to estimate the odds ratios (ORs) and 95% confidence intervals (CIs) for the association between dietary patterns and IFG.

**Results:**

Three major dietary patterns were identified: healthy, Western-like, and CarnFat (Carnivorous-fat). In the fully adjusted model, individuals in the highest tertile of the healthy dietary pattern had a lower likelihood of IFG compared to those in the lowest tertile (OR = 0.68, 95% CI: 0.53–0.88). The second tertile of the healthy pattern was also associated with lower odds of IFG (OR = 0.77, 95% CI: 0.62–0.96). No significant associations were found for the Western-like and CarnFat dietary patterns.

**Conclusion:**

A healthy dietary pattern characterized by high intakes of fruits, vegetables, low-fat dairy, nuts, seeds, olive oil, legumes, fish, and whole grains was associated with a lower risk of IFG. These findings highlight the importance of promoting healthy dietary patterns for the prevention of prediabetes and type 2 diabetes.

## 1 Introduction

Impaired fasting glucose (IFG) refers to a fasting plasma glucose (FPG) level that falls between normal and diabetes cutoff values (1). The expert committee of the American Diabetes Association (ADA) introduced the concept of IFG in 1997, defining it as FPG levels between 110 and 125 mg/dL (2). According to this criterion, the global prevalence of IFG was 5.8% (~298 million individuals) in 2021, and is expected to increase to 6.5% (~414 million individuals) by 2045 (3). However, in 2003, the ADA revised the definition of IFG to encompass FPG levels ranging from 100 to 125 mg/dL (4). Studies utilizing this updated cutoff have reported a higher prevalence of IFG; for instance, nearly one-fourth of adults surveyed in the New York City Health and Nutrition Examination Survey (NYC HANES) were found to have IFG, with the highest prevalence observed among Asians [32.4%; (5)]. Similarly, a population-based study conducted among adults aged 35 to 70 years in Iran reported a prevalence of IFG of 20.61% (6). IFG has been linked with an elevated risk of developing diabetes and experiencing vascular complications (7). Understanding the risk factors associated with IFG and developing individualized management strategies are essential for delaying or preventing its progression and the associated consequences.

Literature suggests the impact of certain dietary patterns on metabolic health, including IFG. A recent cross-sectional study conducted among Chinese men revealed an increased odds of IFG associated with an animal offal-dessert dietary pattern, a decreased likelihood associated with a vegetables-fruits dietary pattern, and no significant association with a white rice-red meat dietary pattern (8). Conversely, another prospective study from the Tehran Lipid and Glucose Study in Iran found no significant association between tertiles of Western, traditional, and healthy dietary patterns and the risk of IFG or prediabetes (9). Given the variations in predominant dietary patterns across different geographical regions and timeframes of investigation, as well as the limited evidence and conflicting findings in the literature, we utilized data of the PERSIAN Kavar cohort study (PKCS) to recognize the major dietary patterns among the included population and to elucidate how these different dietary patterns may influence the odds of IFG.

## 2 Methods

In this cross-sectional study, we used the baseline data from PKCS, a branch of PERSIAN (Prospective Epidemiological Research Studies in IrAN) cohort study conducted in Kavar city, located in the central region of the Fars province in Iran. The PKCS is conducted on 4,997 participants aged 35–70 years. The detailed protocol has been previously published (10). Written informed consent was signed by all participants. Individuals with pre-existing chronic conditions such as malignancies, hepatitis, renal failure, diabetes, hypertension, cardiac diseases, and hyperlipidemia as well as those with FPG levels higher than 125 mg/dL and pregnant women were excluded from the current analysis. The protocol of this study adhered to the Declaration of Helsinki and was approved by the Ethics Committee of Shiraz University of medical sciences, Shiraz, Iran (Code: IR.SUMS.REC.1399.1092).

The data collection involved the use of validated questionnaires, blood sampling, and physical examinations (10, 11). The socio-economic status of the participants was evaluated by the wealth score index (WSI), which considered the assets of their households (10). The FPG levels were measured using Iranian commercial kits (Pars Azmoon) and an auto-analyzer (model BT3000 Plus, Biotecnica^®^, Italy). The IFG was defined as FPG value of 100 to 125 mg/dL (1). Dietary intake over the previous year was assessed using a validated food frequency questionnaire (12). Trained nutritionists queried each participant about the frequency of consuming individual food items on a daily, weekly, monthly, or yearly basis, as well as the quantity consumed based on predetermined portion sizes. Then, these values were converted to grams of food intake per day. Out of 118 food items, 29 food groups were derived with similar nutrient profile to identify major dietary patterns.

Statistical analyses were conducted using SPSS 26 software. The normality of data was tested using skewness, kurtosis, and standard deviation (SD). Then, normal data was reported as mean ± SD and qualitative data as frequency (percentage). Between-group differences were conducted using ANOVA for quantitative data and chi-square test for qualitatives. For extracting dietary patterns, principal component analysis with varimax rotation was used. Kaiser-Meyer-Olkin (KMO) test and Bartlett's test of sphericity were done for assessing the suitability of the sample size for conducting factor analysis and correlations across food groups, respectively. Major dietary patterns were identified using eigenvalues of > 1.5 (13, 14) and scree plot. For assessing the association between dietary patterns and hyperglycemia, we used multivariable logistic regression, and results are reported as odds ratio (OR) and 95% confidence intervals (CI). Age, sex, ethnicity, marital status, education, socioeconomic status, smoking status, alcohol intake, physical activity, body mass index (BMI), and energy were included in the regression model. A two-sided *P* < 0.05 was considered significant.

## 3 Results

**Figure 1:**
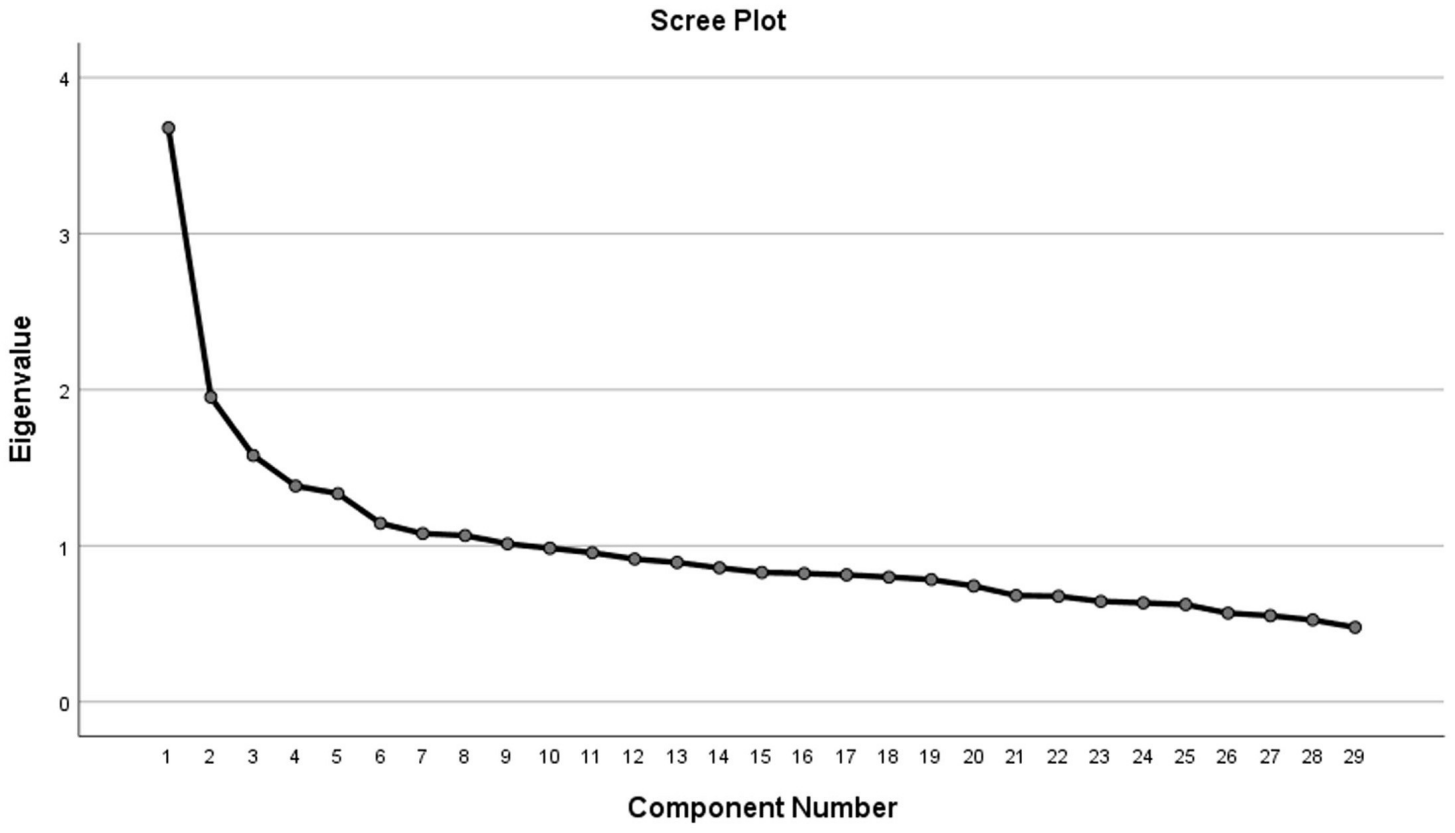
Scree plot from the principal component analysis.

The 29 food groups and their corresponding food items are detailed in Table 1. The suitability of the sample size for conducting factor analysis and inter-item correlations was confirmed by a KMO value of 0.773 and a significant Bartlett's test of sphericity (*P* < 0.001). Subsequently, three major dietary patterns were identified using eigenvalues of > 1.5 and a scree plot (Figure 1). These three dietary patterns include a healthy pattern with high intakes of fruits, vegetables, low-fat dairy, nuts-seeds, dried fruits, olive, legume, pickle, fish, onion-garlic, fresh juice, potato, and whole grain; a Western-like pattern associated with a higher intake of sweetened beverages, proceed meats, refined grains, mayonnaise, pizza, snacks, confectionary-sugar, eggs, high-fat dairy, poultry, salt, and oil, and a lower intake of whole grain; and a CarnFat (Carnivorous-fat) pattern with high factor loading on animal-hydrogenated fat, organ meat, and red meat, and lower levels of soy and oil (Table 2). These dietary patterns explained 24.85% of total variance.

**Table 1:** Food groups and their related food items for deriving dietary patterns.

**Food groups**	**Food item**
Fruits	Cantaloup melon, Honeydew, Watermelon, Apricot, Sour Cherries, Nectarines, Peaches, Prunus, Fresh Berries (Mulberries), Strawberries, Plums, Fresh figs, grapes, Pears, Apples, Kiwifruit, Citrus fruit, Pomegranate, Banana, Persimmon, Dates
Vegetables	Lettuce, Cabbage, Tomato, Cucumbers, Fresh leafy greens, Cooked leafy greens, Eggplant, Celery, Carrots, Bell peppers, Mushroom, Green peas, Green beans, Zucchini, Green peppers
Low-fat dairy	Milk, Yogurt, Cheese, Doogh, kashk
Nuts-seeds	Walnuts, Peanut, Almonds, Hindi, Pistachios, Hazelnuts, Pumpkin watermelon, Sunflower
Dried fruits	Raisins, Dried fruits
Olive	Olive and olive oil
Legume	Bean, Chickpeas, Mung beans, Lentils, dhal, Split peas, Fava beans, Lima beans
Pickle	Pickles, Torshi/Pickled vegetables (in vinegar), Pickled vegetables (in salt water)
Fish	Fish
Onion-Garlic	Onion, Garlic
Fresh juice	Fresh juice
Potato	Potato
Whole grain	Whole wheat bread, Wheat, Oats, Barley, Corn, Traditional Bread
Sweetened beverage	Soda drinks, Non-alcoholic malt beverage, Juice
Proceed meats	Hamburger, Sausages, Kilbasa, Salami
Refined grain	Rice, White wheat bread, Pasta, Noodles, Baguette
Mayonnaise	Mayonnaise
Pizza	Pizza
Snack	Cheese Puffs, Cheeps
Confectionary-Sugar	Cake, Jam, Honey, Chocolate, Crackers, Wafers, Biscuits, Sugar, Rock candy, Noghl
Egg	Egg
High-fat dairy	Ice-cream, Cream, Clotted cream
Poultry	Chicken
Salt	Salt
Oil	Oils
Animal-hydrogenated fat	Margarine, Butter, Hydrogenated fat, Animal fat
Organ meat	Variety meats and by-products (heart, liver, kidney, brain, tongue, tripe, leg, and trotter of sheep)
Red meat	Red meat
Soy	Soybean

**Table 2:** Factor loading matrix for dietary patterns.

**Food groups**	**Healthy pattern**	**Western-like pattern**	CarnFat pattern^*^
Fruits	0.713	0.022	0.062
Vegetables	0.587	0.152	−0.033
Low-fat dairy	0.547	0.093	−0.020
Nuts-seeds	0.540	0.125	0.053
Dried fruits	0.527	−0.131	0.094
Olive	0.471	−0.125	0.000
Legume	0.451	0.211	−0.133
Pickle	0.385	0.197	−0.005
Fish	0.377	0.066	−0.015
Onion-Garlic	0.349	0.080	0.022
Fresh juice	0.328	−0.057	0.158
Potato	0.288	0.285	−0.179
Whole grain	0.276	−0.228	0.246
Sweetened beverage	0.010	0.581	0.237
Proceed meats	−0.103	0.477	−0.014
Refined grain	−0.111	0.468	−0.066
Mayonnaise	0.107	0.453	0.057
Pizza	0.082	0.434	0.037
Snack	0.029	0.394	0.058
Confectionary-Sugar	0.053	0.376	0.223
Egg	0.172	0.358	−0.061
High-fat dairy	0.267	0.323	−0.015
Poultry	0.282	0.303	−0.111
Salt	0.065	0.227	−0.087
Oil	0.074	0.229	−0.632
Animal-hydrogenated fat	−0.035	0.161	0.565
Organ meat	0.172	0.262	0.526
Red meat	0.302	0.176	0.465
Soy	0.200	0.207	−0.308
Percentage of variance explained (%)	11.11	8.22	5.52

^*^Carn stands for Carnivorous. Factor analysis was used.

**Figure 2:**
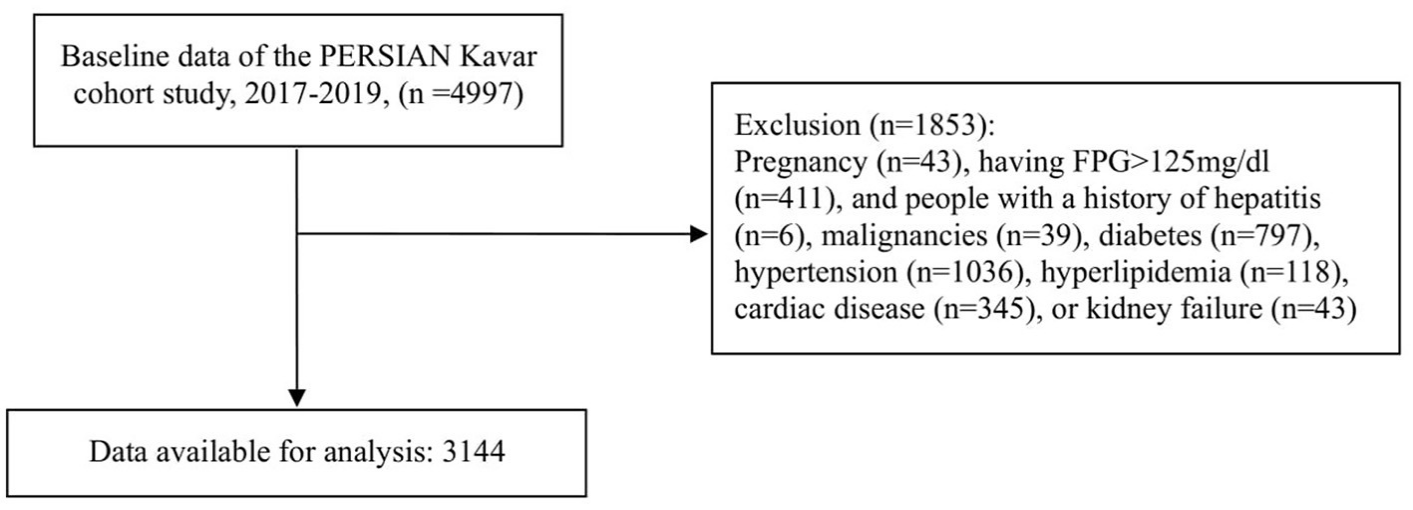
Flowchart of study.

Among the 4,997 participants in the baseline phase of the PKCS, 3,144 subjects (54% men and 46% women with a mean age of 45.75 years) were included in the final analysis (Figure 2), and their characteristics are reported in Table 3. Upon assessing the subjects' features across tertiles of adherence to dietary patterns, it was observed that individuals with greater adherence to the healthy dietary pattern were more likely to be male (*P* < 0.001), of *Persian* ethnicity (*P* < 0.001), have a higher level of education (*P* < 0.001), exhibit a higher socio-economic status (*P* < 0.001), have a higher BMI (*P* < 0.001), and consume more energy (*P* < 0.001). Additionally, there were significant differences in sex, education, smoking, alcohol intake, age, BMI, and energy among the tertiles of Western-like and CarnFat dietary patterns (Table 3).

**Table 3:** Baseline characteristics of the major dietary patterns by factor score tertiles.

**Characteristics**	**Total (*n* = 3,144)**	**Heathy pattern**				**Western-like pattern**				**CarnFat pattern**			
		**T1**	**T2**	**T3**	***P***	**T1**	**T2**	**T3**	***P***	**T1**	**T2**	**T3**	***P***
**Sex, n (%)**													
Men	1,693 (53.8)	507 (48.4)	564 (53.8)	622 (59.4)	**< 0.001**	407 (38.8)	551 (52.6)	735 (70.1)	**< 0.001**	510 (48.7)	568 (54.2)	615 (58.7)	**< 0.001**
Women	1,451 (46.2)	541 (51.6)	484 (46.2)	426 (40.6)		641 (61.2)	497 (47.4)	313 (29.9)		538 (51.3)	480 (45.8)	433 (41.3)	
**Ethnicity, n (%)**													
Persian	2,379 (75.7)	724 (69.1)	790 (75.4)	865 (82.5)	**< 0.001**	802 (76.5)	795 (75.9)	782 (74.6)	0.722	891 (85.0)	805 (76.8)	683 (65.2)	**< 0.001**
Turk	638 (20.3)	289 (27.6)	209 (19.9)	140 (13.4)		210 (20.0)	209 (19.9)	219 (20.9)		117 (11.2)	197 (18.8)	324 (30.9)	
Others	127 (4.0)	35 (3.3)	49 (4.7)	43 (4.1)		36 (3.4)	44 (4.2)	47 (4.5)		40 (3.8)	46 (4.4)	41 (3.9)	
**Education, n (%)**													
Illiterate	753 (24.0)	340 (32.4)	246 (23.5)	167 (15.9)	**< 0.001**	313 (29.9)	264 (25.2)	176 (16.8)	**< 0.001**	202 (19.3)	241 (23.0)	310 (29.6)	**< 0.001**
Elementary	984 (31.3)	307 (29.3)	331 (31.6)	346 (33.0)		312 (29.8)	310 (29.6)	362 (34.5)		319 (30.4)	325 (31.0)	340 (32.4)	
Middle/high school	1,083 (34.4)	323 (30.8)	363 (34.6)	397 (37.9)		310 (29.6)	363 (34.6)	410 (39.1)		410 (39.1)	364 (34.7)	309 (29.5)	
Academic	324 (10.3)	78 (7.4)	108 (10.3)	138 (13.2)		113 (10.8)	111 (10.6)	100 (9.5)		117 (11.2)	118 (11.3)	89 (8.5)	
**Marital status, n (%)**													
Single	104 (3.3)	50 (4.8)	25 (2.4)	29 (2.8)	**< 0.001**	45 (4.3)	26 (2.5)	33 (3.1)	**< 0.001**	33 (3.1)	33 (3.1)	38 (3.6)	0.341
Married	2,908 (92.5)	937 (89.4)	984 (93.9)	987 (94.2)		936 (89.3)	986 (94.1)	986 (94.1)		963 (91.9)	981 (93.6)	964 (92.0)	
Others	132 (4.2)	61 (5.8)	39 (3.7)	32 (3.1)		67 (6.4)	36 (3.4)	29 (2.8)		52 (5.0)	34 (3.2)	46 (4.4)	
**Smoking, n (%)**													
Smoker	590 (18.8)	210 (20.0)	181 (17.3)	199 (19.0)	0.588	95 (9.1)	190 (18.1)	305 (29.1)	**< 0.001**	171 (16.3)	172 (16.4)	247 (23.6)	**< 0.001**
Ex-smoker	224 (7.1)	71 (6.8)	77 (7.3)	76 (7.3)		63 (6.0)	65 (6.2)	96 (9.2)		66 (6.3)	77 (7.3)	81 (7.7)	
Non-smoker	2,330 (74.1)	767 (73.2)	790 (75.4)	773 (73.8)		890 (84.9)	793 (75.7)	647 (61.7)		811 (77.4)	799 (76.2)	720 (68.7)	
**Alcohol intake, n (%)**					0.103				**< 0.001**				**0.001**
Yes	341 (10.8)	111 (10.6)	100 (9.5) 948	130 (12.4)		48 (4.6)	84 (8.0)	209 (19.9)		100 (9.5)	94 (9.0)	147 (14.0)	
No	2,803 (89.2)	937 (89.4)	(90.5)	918 (87.6)		1,000 (95.4)	964 (92.0)	839 (80.1)		948 (90.5)	954 (91.0)	901 (86.0)	
**WSI, n (%)**													
T1	1,139 (36.2)	534 (51.0)	349 (33.3)	256 (24.4)	**< 0.001**	380 (36.3)	375 (35.8)	384 (36.6)	0.069	382 (36.5)	345 (32.9)	412 (39.3)	**0.049**
T2	953 (30.3)	303 (28.9)	363 (34.6)	287 (27.4)		288 (27.5)	327 (31.2)	338 (32.3)		314 (30.0)	339 (32.3)	300 (28.6)	
T3	1,052 (33.5)	211 (20.1)	336 (32.1)	505 (48.2)		380 (36.3)	346 (33.0)	326 (31.1)		352 (33.6)	364 (34.7)	336 (32.1)	
Age (years), mean ± SD	45.75 ± 8.04	45.73 ± 8.33	45.64 ± 7.86	45.88 ± 7.94	0.790	47.55 ± 8.58	45.57 ± 7.71	44.13 ± 7.43	**< 0.001**	44.79 ± 7.75	45.85 ± 8.03	46.61 ± 8.25	**< 0.001**
BMI (kg/m^2^), mean ± SD	26.68 ± 4.69	26.01 ± 4.99	26.83 ± 4.46	27.20 ± 4.54	**< 0.001**	27.21 ± 4.61	26.57 ± 4.78	26.26 ± 4.63	**< 0.001**	27.24 ± 4.69	26.70 ± 4.60	26.10 ± 4.72	**< 0.001**
Activity level (MET-h/week), mean ± SD	41.89 ± 6.83	42.16 ± 6.96	41.50 ± 6.49	42.01 ± 7.01	0.069	41.95 ± 5.89	41.71 ± 6.85	42.01 ± 7.63	0.563	41.51 ± 6.61	41.84 ± 6.71	42.32 ± 7.14	**0.024**
Total energy intake (Kcal), mean ± SD	2,245.06 ± 607.74	1,904.66 ± 457.41	2,204.70 ± 491.15	2,625.81 ± 628.95	**< 0.001**	1,971.71 ± 560.25	2,146.94 ± 450.48	2,616.52 ± 607.79	**< 0.001**	2,179.08 ± 546.17	2,146.89 ± 561.00	2,409.21 ± 674.35	**< 0.001**

BMI, body mass index; HDL-C, high-density lipoprotein cholesterol; T, tertile; WSI, Wealth score index. Between-group differences were assessed using analysis of variance (ANOVA) test for parametric variables and Chi-square test for categorical variables. Bold denotes statistical significance (*P* < 0.05).

Among the included population, 689 subjects (21.9%) had IFG. Table 4 presents the odds ratios of IFG based on the tertiles of dietary pattern scores. In the fully adjusted model, a reduced likelihood of IFG was observed in individuals with the highest adherence to the healthy dietary pattern than persons with the lowest adherence (OR = 0.68, 95% CI: 0.53–0.88, *P* = 0.004). Subjects in the second tertile had also significantly lower odds of IFG compared to the reference (OR = 0.77, 95% CI: 0.62–0.96, *P* = 0.018).

**Table 4:** Odds of impaired fasting glucose according to the tertiles of dietary pattern's scores.

**Models**	**Dietary patterns**			***P trend***
	**T1**	**T2**	**T3**	
**Heathy pattern**				
Event (%)	251 (24.0)	220 (21.0)	218 (20.8)	
Model 1	1.00 (Ref.)	0.84 (0.69–1.04)	0.83 (0.67–1.02)	0.073
Model 2	1.00 (Ref.)	0.77 (0.62–0.96)	0.68 (0.53–0.88)	**0.003**
**Western-like pattern**				
Event (%)	236 (22.5)	211 (20.1)	242 (23.1)	
Model 1	1.00 (Ref.)	0.90 (0.72–1.11)	1.10 (0.88–1.36)	0.407
Model 2	1.00 (Ref.)	0.93 (0.74–1.15)	1.11 (0.87–1.41)	0.429
**CarnFat pattern**				
Event (%)	224 (21.4)	219 (20.9)	246 (23.5)	
Model 1	1.00 (Ref.)	0.95 (0.77–1.18)	1.09 (0.89–1.35)	0.383
Model 2	1.00 (Ref.)	1.01 (0.81–1.25)	1.21 (0.98–1.51)	0.083

CI, confidence interval; OR, odds ratio. Data are reported as ORs and 95% CI which were determined by multivariable logistic regression. Model 1: Adjustment for age and sex; Model 2: Adjustment for age, sex, ethnicity, marital status, education, socioeconomic status, smoking status, alcohol intake, physical activity, body mass index, and energy. The bold font indicates statistical significance (*P* < 0.05).

## 4 Discussion

**Figure 3:**
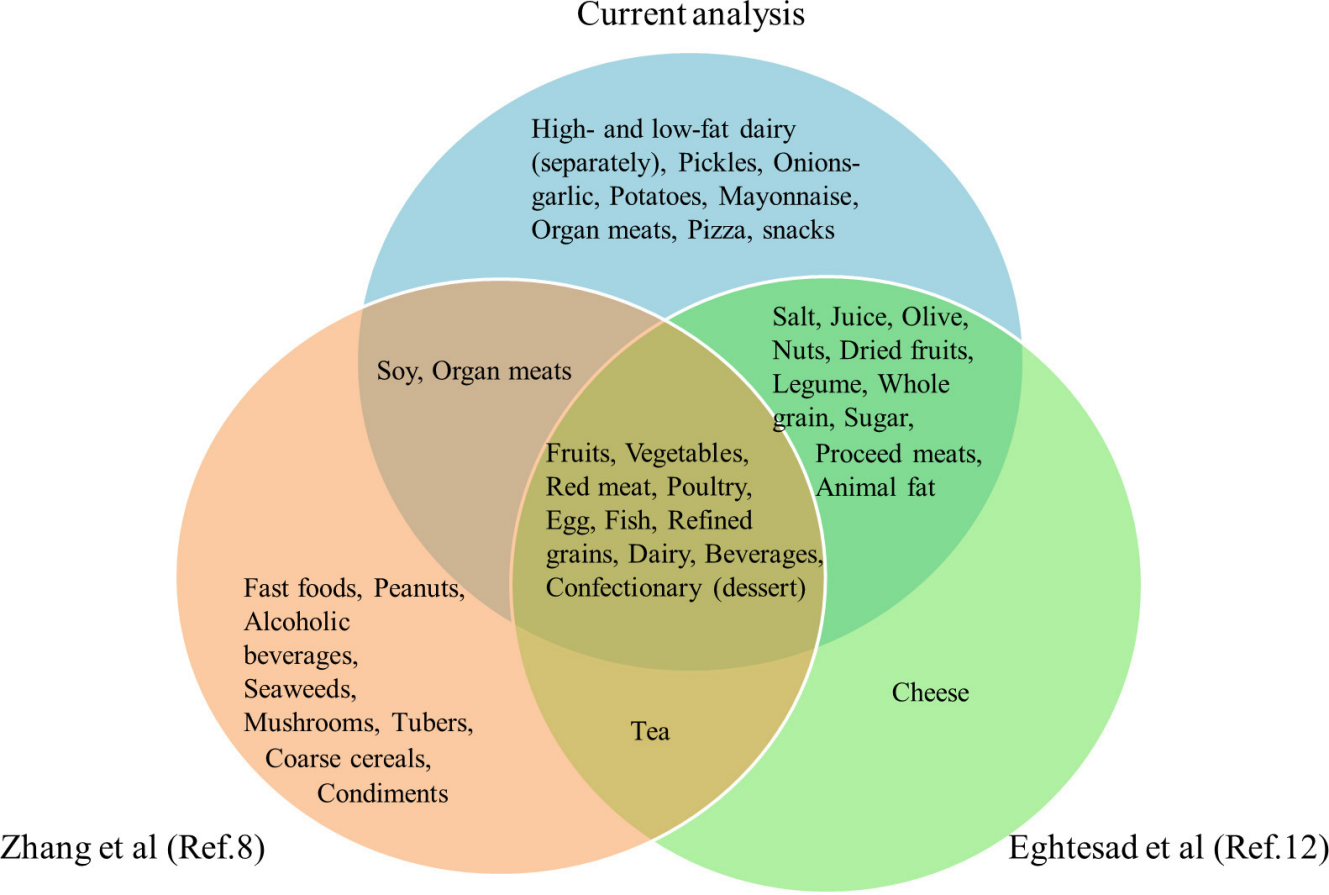
Venn diagram for the comparison of food groups between the current analysis and others.

The three major dietary patterns identified in the current study were categorized as healthy, Western-like, and CarnFat. These dietary patterns were derived from 29 food groups, which is a greater number than those identified in a previous study involving seven centers of the PERSIAN Cohort Study, which derived 23 food groups from the FFQ questionnaire. Notably, this earlier study did not categorize high-fat dairy products, pickles, onions, garlic, potatoes, mayonnaise, organ meats, pizza, and snacks as separate categories, unlike our study (12). The factor loadings for the healthy dietary pattern in this study were largely consistent with those identified in our research; however, the other patterns exhibited significant differences. In another similar study conducted by Zhang et al., three dietary patterns—vegetables-fruits, animal offal-dessert, and white rice-red meat—were extracted from 21 food groups (8). Fast foods, peanuts, alcoholic beverages, seaweeds, mushrooms, tubers, coarse cereals, and condiments were considered as separate categories in that study, whereas they were not distinctly categorized in either our study or the one by Eghtesad et al. (12). A comparison of food groups between the current analysis and these two studies is depicted in Figure 3. Furthermore, our dietary patterns showed considerable alignment with those identified in a previous study by Mirzababaei et al., which examined dietary patterns among overweight and obese Iranian women. This study identified three major patterns: a healthy dietary pattern, including, vegetables, fruits, legumes, nuts, low-fat dairy products, white meat, and olives; a Western-like dietary pattern marked by high levels of fast food, mayonnaise, snacks, high-energy beverages, sweets, cereals, and condiments; and an unhealthy dietary pattern rich in solid fats, high-fat dairy products, red meat, organ meats (such as liver, brain, and kidney), tea and coffee, along with lower consumption of liquid oils (15). The factor loadings in this study across different dietary patterns were largely similar to those observed in our research.

Our findings indicate that subjects in the second and third tertiles of adherence to the healthy dietary pattern exhibited, respectively, 23 and 32% lower odds of IFG, representing a small effect. Limited studies have investigated the association between dietary patterns and IFG. Consistent with our results, Zhang et al. conducted a cross-sectional study on Chinese men, revealing that a vegetables-fruits dietary pattern, characterized by high intake of vegetables and fruits as well as cereals, refined grain, soybean, peanuts, and dairy products, was negatively associated with the odds of IFG, defined as FPG levels between 110 and 126 mg/dL (8). In another prospective study on 904 Iranian subjects, a healthy dietary pattern (high load of vegetables and their oils, fresh and dried fruits, low-fat dairy, and nuts and seeds) was inversely associated with 3-year changes in FPG, fasting insulin, 2 h-serum glucose, and homeostasis model assessment of insulin resistance in linear regression models. However, no relationship was found between tertile categories of the healthy dietary pattern and IFG (FPG levels of 100–126 mg/dL), impaired glucose tolerance (IGT, 2-h serum glucose levels of 140–199 mg/dL), prediabetes (defined as having either IFG or IGT, or β-cell dysfunction in multivariable logistic regression models. Indeed, while a healthy dietary pattern was associated with reduced FPG levels, it did not significantly diminish the risk of developing IFG (9). This dietary pattern is linked to adequate micronutrient intake (16). The beneficial effects of a healthy dietary pattern on IFG may be attributed to its high content of vitamins, minerals, antioxidants, anti-inflammatory compounds, phytochemicals, fiber, monounsaturated fats, and ω-3 fatty acids, all of which had beneficial effects on IFG (17–20). Discrepancies among studies regarding the effects of healthy patterns on IFG and diabetes prevention may arise from differences in study populations, the specific factors loaded in each study's dietary pattern, and variations in disease threshold definitions.

Here, we did not observe any association between Western-like and CarnFat dietary patterns and the odds of IFG. This finding is consistent with some (9, 21) previous studies but contrasts with a body of literature that establishes links between Western diets and some metabolic disorders, like IFG, insulin resistance, and diabetes (8, 22, 23). In a study by Walsh *et al*. involving 209 subjects aged 60–65 (28 of whom had IFG), no association was reported between the highest tertile of Western diet score and IFG (21). Similarly, Doostvandi *et al*. found no association between the Western dietary pattern (fast foods, salty snacks, mayonnaise, soft drinks, confectionary, and organ meats) and FPG in linear model, nor with the incidence of IFG, prediabetes, β-cell dysfunction, and hyperinsulinemia in the logistic models. However, they detected a significant relationship between the Western dietary pattern and IGT development (9). One potential explanation for this observation could be the possibility that the mechanisms underlying the effects of a Western diet on IFG may differ from those related to IGT (21). A previous meta-analysis of randomized controlled trials reported no significant effect of red meat consumption on FPG, fasting insulin, postprandial insulin, insulin sensitivity, homeostatic model assessment of insulin resistance (HOMA-IR), hemoglobin A1c, pancreatic beta-cell function, or glucagon-like peptide-1. The authors suggested that discrepancies among studies regarding the association between red meat and diabetes risk may be attributable to residual confounding factors associated with higher red meat consumption (24). Furthermore, the existing data concerning the role of saturated fatty acids in diabetes remain inconsistent (25, 26). Variations in study design and unadjusted lifestyle factors may contribute to the contradictory findings observed between Western-style and CarnFat dietary patterns in relation to diabetes. Additionally, genetic predisposition and racial ancestry have been reported to interact with the impact of the Western dietary pattern on the risk of diabetes and metabolic syndrome (27, 28). In current study, we were unable to assess the duration of dietary exposure; this limitation should be addressed in future prospective studies aimed at evaluating the effects of dietary patterns on glucose metabolism.

Our study had several limitations. We could not determine the direction of the association due to the cross-sectional design of the current study. Furthermore, data related to insulin levels, hemoglobin A1C, and IGT were not available in our database, preventing us from assessing the effects of dietary patterns on these variables. Additionally, despite adjusting for several confounding factors, some unmeasured or unknown variables may still influence the reported associations. Future prospective longitudinal studies with large sample size are needed to clarify the effects of various dietary patterns on glucose metabolism more comprehensively.

In conclusion, healthy dietary pattern, which was characterized with high intake of fruits, vegetables, low-fat dairy, nuts-seeds, dried fruits, olive, legume, pickle, fish, onion-garlic, fresh juice, potato, and whole grain, was associated with lower odds of IFG. However, no association was found for Western-like and CarnFat dietary patterns.

## Data Availability

The raw data supporting the conclusions of this article will be made available by the authors, without undue reservation.
